# Delayed Migration of an Amplatzer PFO Occluder to the Infrarenal Abdominal Aorta: Successful Endovascular Snare Retrieval

**DOI:** 10.3390/reports9010068

**Published:** 2026-02-25

**Authors:** Fulvio Cacciapuoti, Elisa Rusciano, Rodolfo Nasti, Mafalda Esposito, Ciro Mauro

**Affiliations:** 1 Division of Cardiology, “Antonio Cardarelli” Hospital, 80131 Naples, Italy; 2 Postgraduate School of Cardiology, “Federico II” University, 80131 Naples, Italy; 3 Division of Internal Medicine, “Betania” Hospital, 80131 Naples, Italy

**Keywords:** Amplatzer device, patent foramen ovale, device migration, abdominal aorta, interventional radiology

## Abstract

**Background and Clinical Significance:** Embolization of septal occluder devices after patent foramen ovale (PFO) closure is uncommon but potentially serious, as migrated devices may lodge in the arterial system and require urgent management. Cross-sectional imaging may reveal delayed migration incidentally, and endovascular snare retrieval represents a minimally invasive first-line strategy in stable patients. **Case Presentation:** An 18-year-old woman presented with acute abdominal pain one month after percutaneous PFO closure performed for preventive purposes in the setting of migraine with visual aura. Contrast-enhanced computed tomography (CT), obtained for suspected intra-abdominal bleeding, demonstrated hemoperitoneum from a hemorrhagic ovarian cyst and incidentally identified the Amplatzer occluder lodged in the infrarenal abdominal aorta with preserved renal artery patency. Transthoracic echocardiography confirmed device absence at the interatrial septum. Endovascular retrieval was performed via right common femoral artery access (5 Fr upsized to 12 Fr) using a 20 mm snare system, with successful removal of the device through the introducer and no intra-procedural complications. **Conclusions:** Delayed migration of a PFO occluder can be detected incidentally during evaluation for unrelated symptoms. In hemodynamically stable patients, transfemoral endovascular snare capture and re-sheathing through a large-bore introducer can achieve safe and effective device retrieval while preserving aorto-iliac patency.

Percutaneous closure of PFO has become a widely accepted therapeutic strategy in selected patients, particularly for secondary prevention of cryptogenic stroke and, in some cases, for the management of migraine with aura [1]. The introduction of transcatheter occlusion devices, such as the Amplatzer occluder, has significantly reduced procedural invasiveness and is associated with high success rates and a low incidence of complications.

Despite the overall safety of this technique, device-related complications may occur. Among these, device migration or embolization represents a rare but potentially serious event, with reported embolization rates around 0.55% in experienced series [2]. Migration can occur early or in a delayed fashion and may involve various vascular territories, including the cardiac chambers, pulmonary arteries, or the systemic arterial circulation [3]. When migration occurs, prompt identification is crucial, as the presence of a foreign body within the arterial system may lead to vascular obstruction, ischemic complications, or embolic events.

Imaging plays a central role in the diagnosis of device migration, often allowing incidental detection during investigations performed for unrelated clinical indications. Advances in endovascular techniques have made percutaneous retrieval the preferred treatment in most cases, avoiding open surgical intervention.

An 18-year-old woman presented to the emergency department with acute-onset abdominal pain. Her medical history was notable for percutaneous closure of a PFO using an Amplatzer occluder performed approximately one month earlier at another institution. Percutaneous PFO closure had been performed at an outside center for preventive purposes in the setting of a history of migraine with visual aura; no prior history of cryptogenic stroke was documented in the available records. No other relevant comorbidities were reported.

On admission, the patient was hemodynamically stable, alert, and oriented. Physical examination revealed a soft, mildly tender abdomen without signs of peritonitis. Laboratory tests showed mild anemia (hemoglobin 11.5 g/dL) and elevated D-dimer levels, while coagulation parameters and cardiac biomarkers were within normal limits. Formal hemolysis markers (lactate dehydrogenase, haptoglobin, indirect bilirubin, reticulocyte count) were not available in the recorded emergency laboratory panel; clinically, no features suggestive of hemolysis were observed during hospitalization. Serial hemoglobin measurements remained stable and no jaundice or dark urine was observed.

Given persistent abdominal pain, contrast-enhanced CT of the abdomen (including the aorto-iliac axis) was performed. Imaging demonstrated hemoperitoneum secondary to a hemorrhagic ovarian cyst, with blood collection in the Douglas pouch and peritoneal recesses. Incidentally, CT also revealed migration of the Amplatzer device (Abbott Cardiovascular, Chicago, Illinois, USA) into the infra-renal abdominal aorta, with preserved patency of the renal arteries and no evidence of vascular obstruction or ischemia (Figure 1).

Furthermore, transthoracic echocardiography performed on admission confirmed the absence of the Amplatzer device at the interatrial septum and demonstrated a residual left-to-right shunt across the atrial septum (Figure 2).

As no indication for urgent gynecological surgical intervention was identified and the patient remained clinically stable, endovascular retrieval of the migrated device was planned.

The procedure was performed under local anesthesia via right common femoral artery access. Diagnostic aortography confirmed the presence of the device lodged within the infra-renal abdominal aorta, without impairment of aorto-iliac flow. Right common femoral arterial access was obtained and a 5 Fr introducer was placed. Aortography with a multipurpose catheter confirmed the device in the infrarenal aorta with preserved renal artery patency. After advancing an Amplatz guidewire, the access was upsized to a 12 Fr introducer using progressive fascial dilators. A 20 mm snare (lasso) catheter was advanced under fluoroscopy, looped around the occluder, and tightened to secure the device before controlled re-sheathing into the 12 Fr introducer and retrieval (Figure 3).

In-hospital follow-up included completion angiography confirming preserved aorto-iliac patency and post-procedure ultrasound documenting patency of the common femoral artery and distal vessels. Further elective assessment (transesophageal echocardiography and/or transcranial Doppler) and re-evaluation at the implanting center were recommended; however, long-term radiologic follow-up beyond discharge was not available in the accessible records.

The post-procedural course was uneventful. Serial clinical and laboratory evaluations showed stable hemoglobin levels and progressive resolution of abdominal symptoms. Subsequent cardiological assessment did not reveal urgent findings, and antiplatelet therapy was temporarily withheld. The patient was discharged in good general condition with recommendations for outpatient cardiological and gynecological follow-up.

Percutaneous closure of PFO using occluder devices is a well-established procedure with high technical success rates and a low incidence of complications. Nevertheless, device-related adverse events have been reported, including device-related thrombus, residual shunting, arrhythmias, and, more rarely, migration or embolization [4]. Although uncommon, PFO closure-device embolization/migration has been reported at rates around 0.6% in institutional series and may represent a potentially serious complication requiring prompt recognition and appropriate management [5].

Migration may occur early, usually related to undersizing of the device, inadequate septal rims, or suboptimal positioning, but delayed migration has also been described [6]. Late or very late embolization is particularly insidious, as patients may remain asymptomatic and the complication may be incidentally discovered during imaging performed for unrelated clinical indications [7]. Reported embolization sites include cardiac chambers, pulmonary arteries, and, less frequently, the systemic arterial circulation, such as the abdominal aorta [8].

In the present case, the migrated Amplatzer device was incidentally identified during CT performed for evaluation of acute abdominal pain caused by hemoperitoneum secondary to a hemorrhagic ovarian cyst. Similar incidental diagnoses have been described in the literature, underscoring the pivotal role of cross-sectional imaging in detecting unexpected device-related complications [9]. The preserved patency of the renal and iliac arteries allowed careful procedural planning and avoided emergent surgical intervention.

Management of migrated occluder devices depends on their anatomical location, the patient’s clinical status, and institutional expertise. Endovascular retrieval has become the preferred first-line approach in most cases, offering a minimally invasive and highly effective alternative to open surgical removal. Snare-assisted techniques using large-bore introducer sheaths have been successfully employed even in large-caliber vessels such as the abdominal aorta. Surgical retrieval is generally reserved for cases in which percutaneous techniques fail or when migration results in hemodynamic instability or organ ischemia [10]. Retrieval strategy depends on occluder geometry. Septal/PFO occluders consist of two discs connected by a waist; depending on device design, the proximal and distal discs may be equal or different in diameter, and a central hub/screw (‘hook end’) may provide a stable snaring target [11]. When an embolized device lies obliquely against the arterial wall, gentle catheter manipulation is often required to align the device coaxially, expose the hub or a free strut, and allow controlled re-sheathing into a large-bore introducer, minimizing the risk of intimal trauma [12].

Large-bore femoral arterial access and foreign-body retrieval may be complicated by bleeding, hematoma, pseudoaneurysm, dissection, thrombosis/embolization, and, rarely, aortic wall injury during manipulation and re-sheathing. In our case, arteriotomy closure was performed with a suture-based closure device (ProGlide) [13]. Post-procedure ultrasound confirmed patency of the common femoral artery and distal vessels.

It is worth noting that, in our patient, PFO closure had been performed for preventive purposes in the setting of migraine with visual aura. This reinforces the importance of careful patient selection and follow-up, particularly when indications are outside established stroke-prevention pathways.

Finally, this case emphasizes the importance of post-procedural surveillance following PFO closure. Although standardized follow-up protocols are lacking, awareness of potential delayed complications is crucial. Early recognition of device migration allows timely intervention and reduces the risk of serious adverse events.

Migration of an Amplatzer occluder is a rare but potentially serious complication of percutaneous PFO closure. Delayed migration may remain clinically silent and be incidentally detected during imaging performed for unrelated conditions. Prompt recognition and appropriate management are essential to prevent vascular complications. Endovascular retrieval represents a safe and effective treatment option and should be considered the first-line approach in stable patients. This case highlights the importance of imaging assessment and a multidisciplinary approach in ensuring timely diagnosis and optimal management of device-related complications.

## Figures and Tables

**Figure 1:**
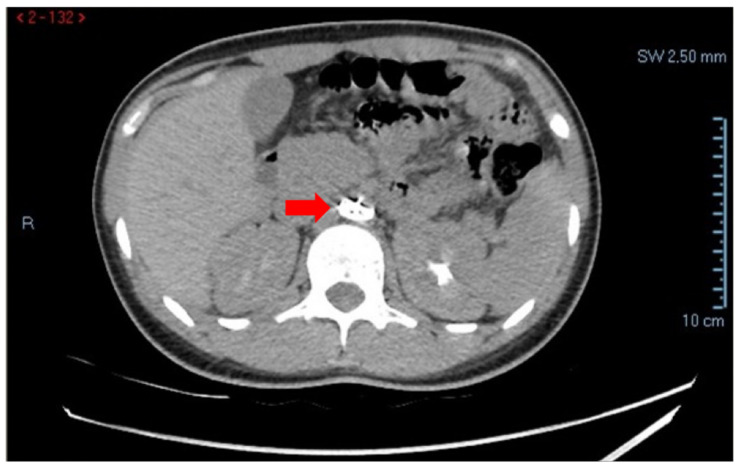
Contrast-enhanced CT of the abdomen demonstrating migration of the Amplatzer occluder into the infra-renal abdominal aorta (arrow), incidentally detected during evaluation for acute abdominal pain.

**Figure 2:**
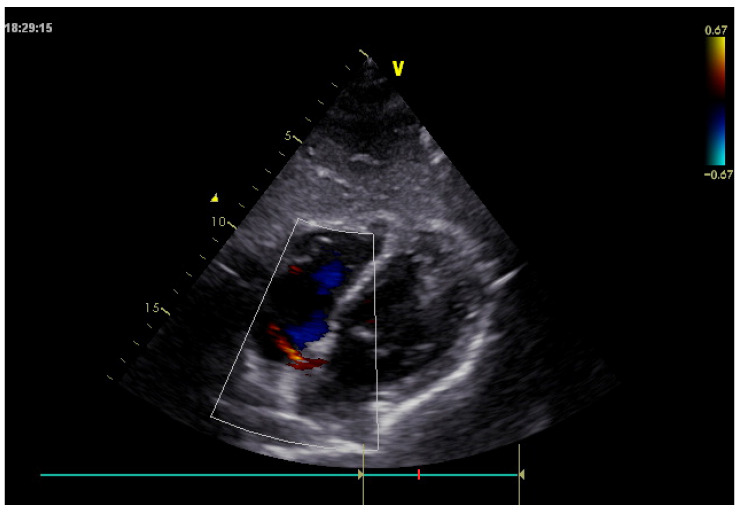
Transthoracic echocardiography showing absence of the Amplatzer device at the interatrial septum and evidence of a residual left-to-right shunt across the atrial septum.

**Figure 3:**
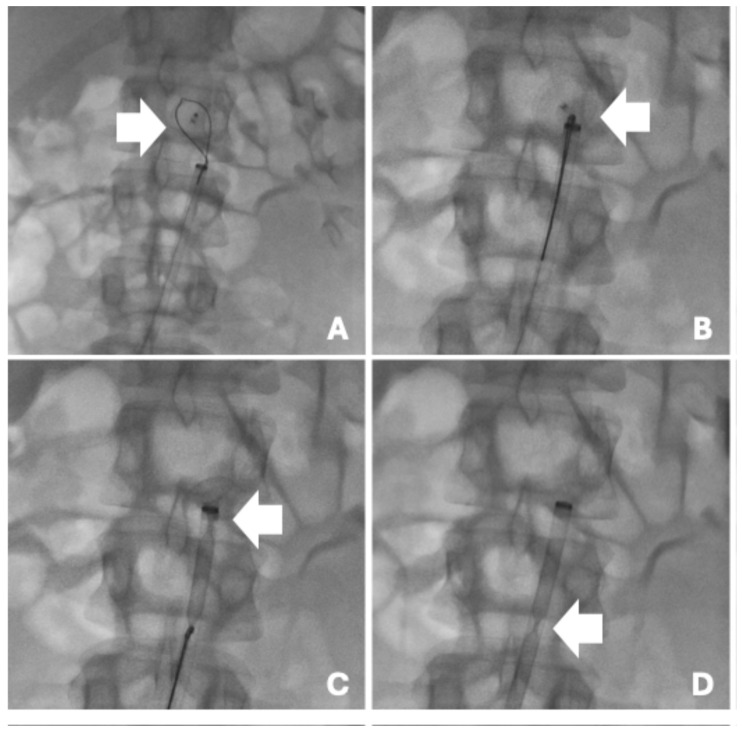
Stepwise endovascular retrieval of the migrated Amplatzer PFO occluder (Abbott Cardiovascular, Chicago, Illinois, USA) from the infrarenal abdominal aorta. (**A**) Snare system positioned around the device with initial engagement (arrow). (**B**) Device secured on the snare and aligned coaxially within the aortic lumen (arrow). (**C**) Advancement/positioning of the large-bore introducer to facilitate re-sheathing of the captured device (arrow). (**D**) Controlled re-sheathing/withdrawal of the device through the introducer (arrow), enabling complete percutaneous removal.

## Data Availability

The original contributions presented in this work are included in the article. Further inquiries can be directed to the corresponding author.
